# Comparison of the Effectiveness and Safety of 5% Cysteamine and 2% Kojic Acid Creams in the Treatment of Melasma in Indian Adult Females

**DOI:** 10.7759/cureus.81330

**Published:** 2025-03-28

**Authors:** Raji Patil, Dhiraj Dhoot, Ashwin Balasubramanian, Saiprasad Patil, Hanmant Barkate

**Affiliations:** 1 Dermatology, Mascot Spincontrol India Pvt. Ltd., Mumbai, IND; 2 Global Medical Affairs, Glenmark Pharmaceuticals Limited, Mumbai, IND

**Keywords:** cysteamine, efficacy, india, kojic acid, melasma, mmasi, safety

## Abstract

Introduction: Hydroquinone, used alone or in combination with a retinoid and corticosteroid, is the preferred first-line treatment for melasma. However, it is associated with side effects such as irritation, erythema, dryness, and exogenous ochronosis. As a result, alternative agents like kojic acid, tranexamic acid, and cysteamine are increasingly being prescribed to minimize these adverse effects.

Aim: This study compared the effectiveness, safety, and tolerability of 5% cysteamine cream with 2% kojic acid cream in Indian adult females with melasma.

Methods: This prospective, randomized, open-label, comparative clinical study involved 72 adult females with facial melasma, who were randomized 1:1 into two groups and instructed to apply either 5% cysteamine or 2% kojic acid cream once daily at night for 16 weeks. The primary endpoints were improvement in modified Melasma Area Severity Index (mMASI) scores, melanin content, dermatological assessment of cosmetic acceptability, and a subject questionnaire on product characteristics and acceptability.

Results: At week 16, the cysteamine group showed a numerically greater mean percentage reduction in mMASI scores (12.25% vs. 10%) and melanin content in lesions by the Mexameter (5.13% vs. 4.81%) (Courage + Khazaka Electronic, Cologne, Germany) from baseline compared to the kojic acid group, though the differences were not statistically significant. No abnormal clinical or functional signs were reported during the study.

Conclusion: Five percent cysteamine cream demonstrated comparable effectiveness to 2% kojic acid cream in melasma patients, with the cysteamine group showing a numerical superiority in mMASI scores and Mexameter readings. Both treatments were well tolerated with no adverse effects. Therefore, 5% cysteamine cream is a valuable addition to the medical management of melasma.

## Introduction

Melasma is a common, acquired, and chronic hyperpigmentation disorder that primarily affects individuals with Fitzpatrick skin types III-VI. It significantly impacts quality of life, causing emotional and psychosocial distress [1-3]. Traditionally, the standard treatment for melasma has involved skin-lightening agents like hydroquinone (HQ), either used alone or in combination with retinoic acid and corticosteroids, known as triple combination therapy (TC) [4]. While effective, these treatments are often associated with adverse effects such as erythema, allergic or irritant contact dermatitis, skin thinning, and telangiectasia. Moreover, prolonged use of HQ-based products for over six months may also lead to exogenous ochronosis. Due to safety concerns, HQ is contraindicated during pregnancy and breastfeeding because of insufficient supporting evidence [5-10].

These potential adverse effects and contraindications have prompted the search for alternative therapies. Non-HQ treatments, such as tranexamic acid (TA), azelaic acid (AA), cysteamine, kojic acid, zinc sulfate, vitamin C, glutathione, carotenoids, and various other antioxidants, have emerged as effective options [11,12]. While both kojic acid and cysteamine are widely prescribed, there is a lack of clinical trials directly comparing their effectiveness and safety.

To the best of our knowledge, this is the first clinical study to compare the clinical effectiveness of 5% cysteamine and 2% kojic acid in Indian adult females with melasma.

## Materials and methods

Study design

This randomized, open-label, comparative clinical study was conducted at Mascot Spincontrol India Pvt. Ltd., Lower Parel, Mumbai, Maharashtra, India, between October 2023 and May 2024. It adhered to the Declaration of Helsinki and was approved by the Ethos Institutional Ethics Committee (Reg. no. IEC/GN/2023/1054). This study was registered with the Clinical Trials Registry India (CTRI/2023/10/058850). Written informed consent was obtained from all participants prior to enrollment, and all data were anonymized.

Participant eligibility criteria

A total of 72 female patients, aged 18 years and older, with facial melasma were included in the study. Participants were selected under the supervision of the investigator. Exclusion criteria included a history of endocrine diseases, pregnancy or breastfeeding, corticosteroid treatment within six months prior to the study, use of oral contraceptives within six months, or any other skin diseases.

Interventions

Patients were randomized in a 1:1 ratio into two groups using a computer-generated block randomization method with a block size of four. Allocation concealment was ensured using sequentially numbered, opaque, sealed envelopes, which were opened by a study coordinator not involved in the outcome assessments after patient enrollment. Patients were instructed to apply either 5% cysteamine cream (Cysteo™, Glenmark Pharmaceuticals Limited, Mumbai, India) or 2% kojic acid cream with Vitamin C (Enshine®, Leeford Healthcare Ltd, Ludhiana, India) once daily at night for 112 days according to their assigned group. Patients in the cysteamine group were advised to apply the cream on an unwashed face. If the face had been washed, a waiting period of 45 minutes to one hour was recommended before applying the product. After 15 minutes of exposure, patients were instructed to wash off the cream with a cleanser and then moisturize. Patients in the kojic acid group were asked to leave the cream on overnight. All patients were provided broad-spectrum sunscreen and instructed to apply and reapply it every 2-3 hours during the day throughout the study period.

Clinical assessment

To reduce inter-rater variability, all modified Melasma Area Severity Index (mMASI) assessments were performed by the same dermatologist throughout the study. Clinical evaluations were performed at baseline, week 2, week 4, week 8, and week 16 for all patients. Effectiveness was assessed based on mMASI scores and Mexameter readings.

The mMASI score is a "reliable index used to quantify the severity of melasma and any changes during therapy." It is calculated by evaluating four facial regions (forehead, right malar, left malar, and chin), each scored for the area of involvement (A) on a scale of 0-6 and darkness (D) and homogeneity (H) on a scale of 0-4. The final score is calculated using the following formula:

mMASI = 0.3(F(D+H)A) + 0.3(RM(D+H)A) + 0.3(LM(D+H)A) + 0.1(C(D+H)A). 

The total mMASI score ranges from 0 to 24 (unitless index), with higher scores indicating more severe melasma [13]. The Mexameter® MX 18 (Courage + Khazaka Electronic, Cologne, Germany) was used to measure melanin content in melasma lesions and adjacent normal skin, expressed in arbitrary units (AU), at baseline and follow-up visits at 2 and 4 months [14]. The difference in melanin content between each lesion and surrounding healthy skin was calculated, and the mean difference was used for statistical analysis.

Patients’ views on treatment effectiveness and product acceptability were assessed using a self-evaluation questionnaire. This questionnaire consisted of eight items related to the product's physical characteristics and tolerability profile (itching, burning, and irritation), rated on a four-point Likert scale (1, completely agree; 2, somewhat agree; 3, somewhat disagree; 4, completely disagree), as provided in Appendix A.

Safety was evaluated by monitoring clinical signs (erythema, edema, dryness, peeling, scaling) by the dermatologist and functional signs (itching, tingling) reported by the patients on a scale from 0 (none) to 3 (severe), at baseline, week 2, week 4, week 8, and week 16. The study flow is illustrated in the Consolidated Standards of Reporting Trials (CONSORT) diagram inFigure 1.** **

**Figure 1:**
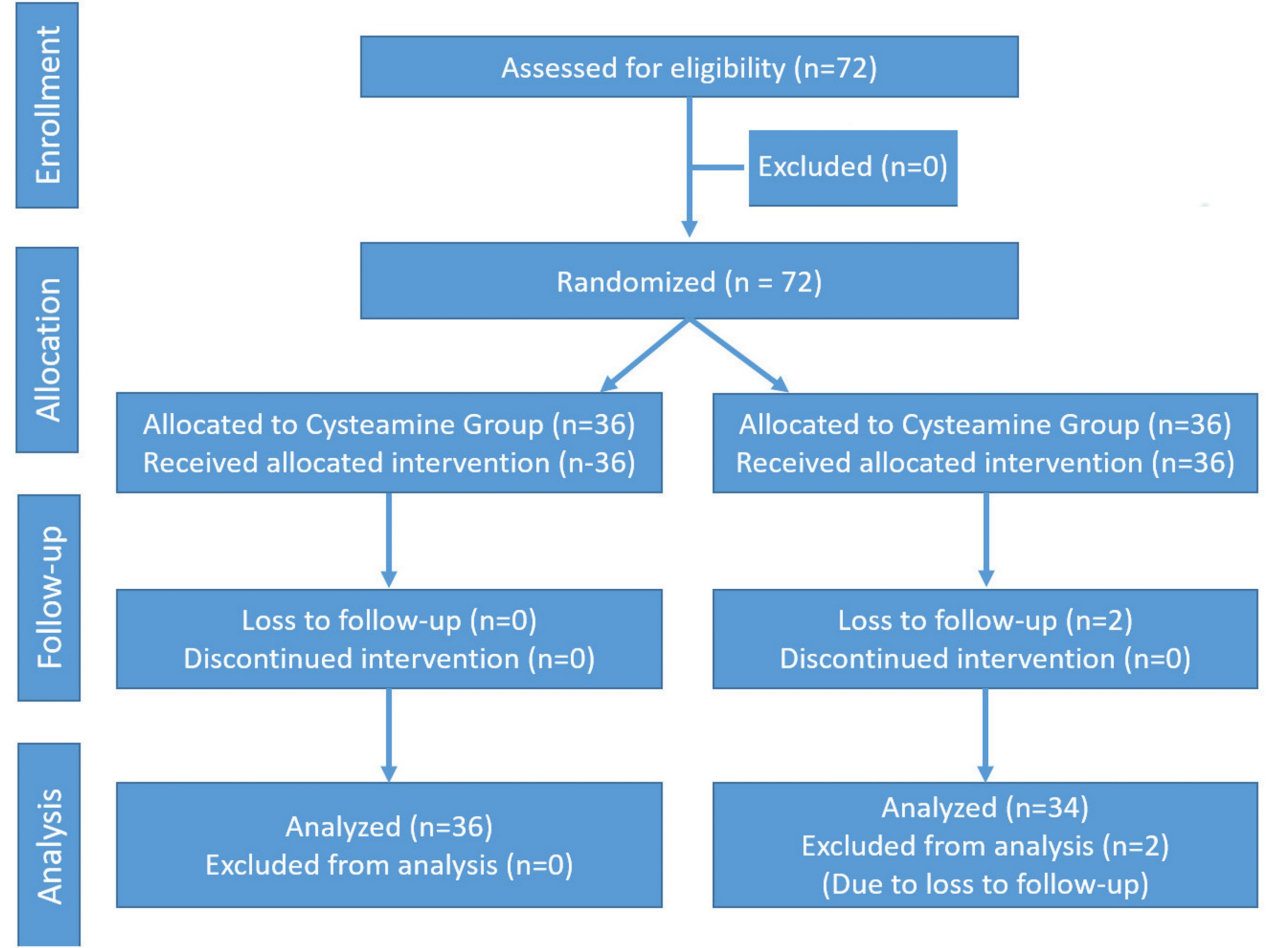
CONSORT diagram of the study flow.

Effectiveness and safety endpoints

The primary endpoint of the study was the improvement in mean mMASI score, as assessed by a dermatologist on days 14, 28, 56, and 112, compared to baseline. The secondary endpoints included the following: (a) the mean reduction in melanin content of pigmented spots, assessed through Mexameter on days 0, 14, 28, 56, and 112, compared to baseline; (b) dermatological assessment of cosmetic acceptability on days 0, 14, 28, 56, and 112; and (c) patient-reported questionnaire on effectiveness, product characteristics, and product acceptability on days 14, 28, 56, and 112.

Statistical analysis

The sample size of 72 participants was determined based on feasibility and was consistent with sample sizes commonly used in comparative dermatology studies of similar duration and design [15,16]. Continuous data were expressed as mean ± standard deviation (SD), and categorical data as number (percent). The Shapiro-Wilk test was used to assess the normality of distributions at a 1% significance level. Statistical comparisons of continuous parameters between follow-up visits and baseline were made using the Wilcoxon-ranked signed test, while between-group comparisons were made using the Student t-test or Mann-Whitney U test at a 5% significance level. Categorical variables were evaluated using the chi-squared test. Statistical analysis was conducted using SPSS for Windows, Version 16.0 (Released 2007; SPSS Inc., Chicago, IL, United States) and Microsoft Excel 2016 (Microsoft Corp., Redmond, WA, United States).

## Results

Demographic and disease characteristics

A total of 72 patients were enrolled, with 36 patients in each group. Two patients from the kojic acid group were lost to follow-up and excluded from the final analysis. The baseline characteristics of both groups are given in Table 1.

**Table 1:** Demographic and disease characteristics of patients at baseline. mMASI: modified Melasma Area Severity Index, AU: arbitrary units.

Parameter	Cysteamine group (n = 36)	Kojic acid group (n = 34)
Age (years, mean ± SD)	40.1 ± 6.6	44.1 ± 5.5
mMASI scores (unitless, mean ± SD)	7.13 ± 2.73	7.76 ± 3.08
Mexameter findings (AU, mean ± SD)	468.55 ± 100.71	468.00 ± 84.96

Effectiveness analysis

**Figure 2:**
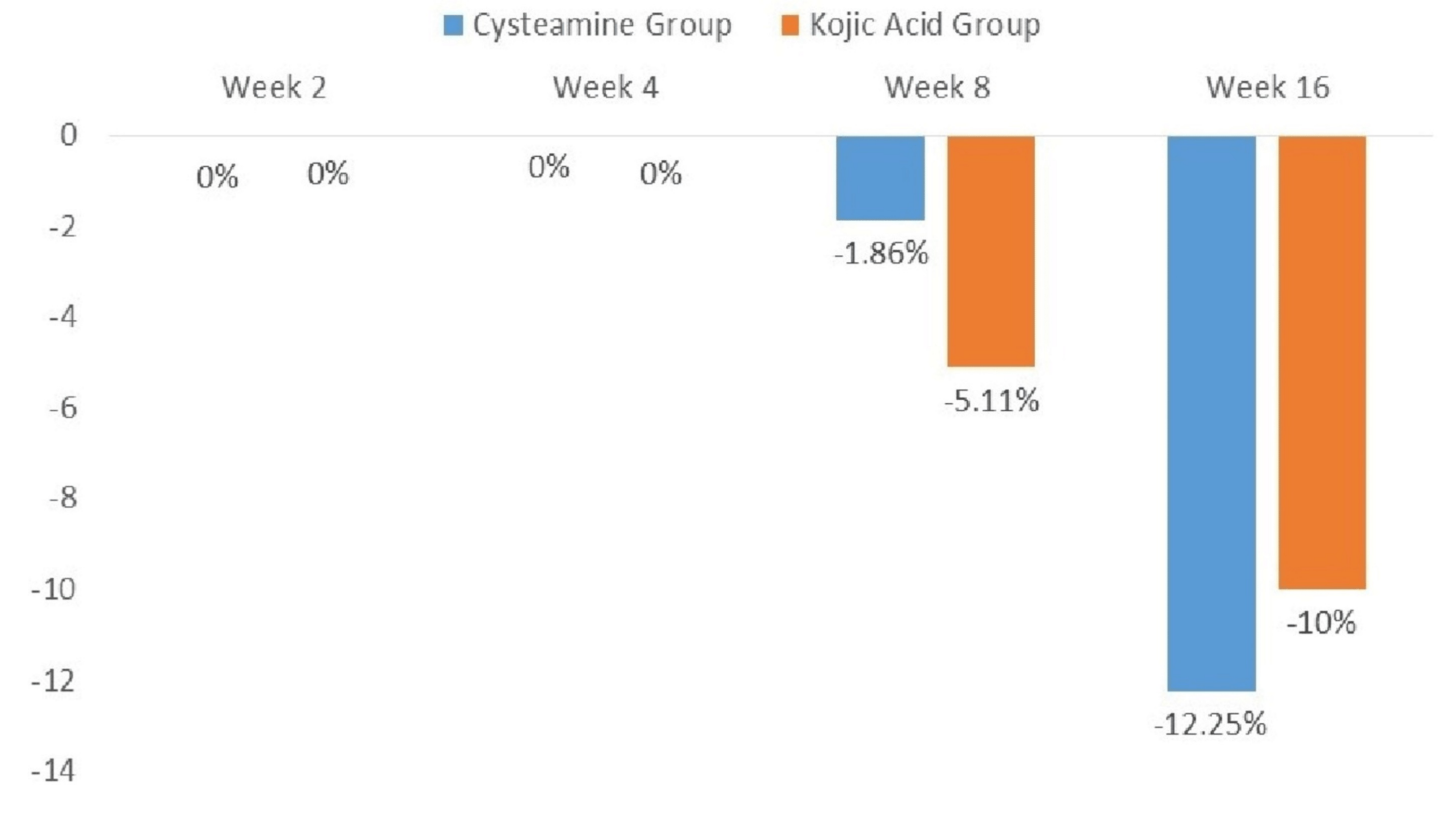
Percentage change in mean mMASI score compared to baseline.

There was a significant decrease in mMASI scores by the end of the study period (week 16) in both groups. The mean mMASI scores at baseline and at each follow-up visit for both groups are shown in Table 2 and Figure 2. In the cysteamine group, the mMASI score at week 16 was reduced by 0.87 (12.25%) compared to baseline (6.32 ± 2.55 vs. 7.13 ± 2.73; p < 0.001). A similar improvement was observed in the kojic acid group, where the mMASI score was reduced by 0.78 (10%) at week 16 compared to baseline (6.99 ± 3.22 vs. 7.76 ± 3.08; p < 0.001), as shown inFigure 2.

**Table 2:** Change in mMASI scores in both groups during the study period. ^+^Wilcoxon signed-rank test, statistically at significant p < 0.05. ^++^Wilcoxon signed-rank test, highly significant at p < 0.001. mMASI: modified Melasma Area Severity Index.

Visit	Cysteamine group (n = 36)	Kojic acid group (n = 34)
Baseline (mMASI score, mean ± SD)	7.13 ± 2.73	7.76 ± 3.08
Week 2 (mMASI score, mean ± SD)	7.13 ± 2.73	7.76 ± 3.08
Week 4 (mMASI score, mean ± SD)	7.13 ± 2.73	7.76 ± 3.08
Week 8 (mMASI score, mean ± SD)	7.06 ± 2.78	7.373 ± 3.02^+^
Week 16 (mMASI score, mean ± SD)	6.32 ± 2.55^++^	6.99 ± 3.22^++^

At week 16, the cysteamine group showed a numerically greater mean reduction in mMASI score (0.87, 12.25%) compared to the kojic acid group (0.78, 10%) from baseline, though the difference was not statistically significant. Seventeen (47%) patients in the cysteamine group and 13 (38%) in the kojic acid group reported improvement in melasma severity by the end of the study.

**Figure 3:**
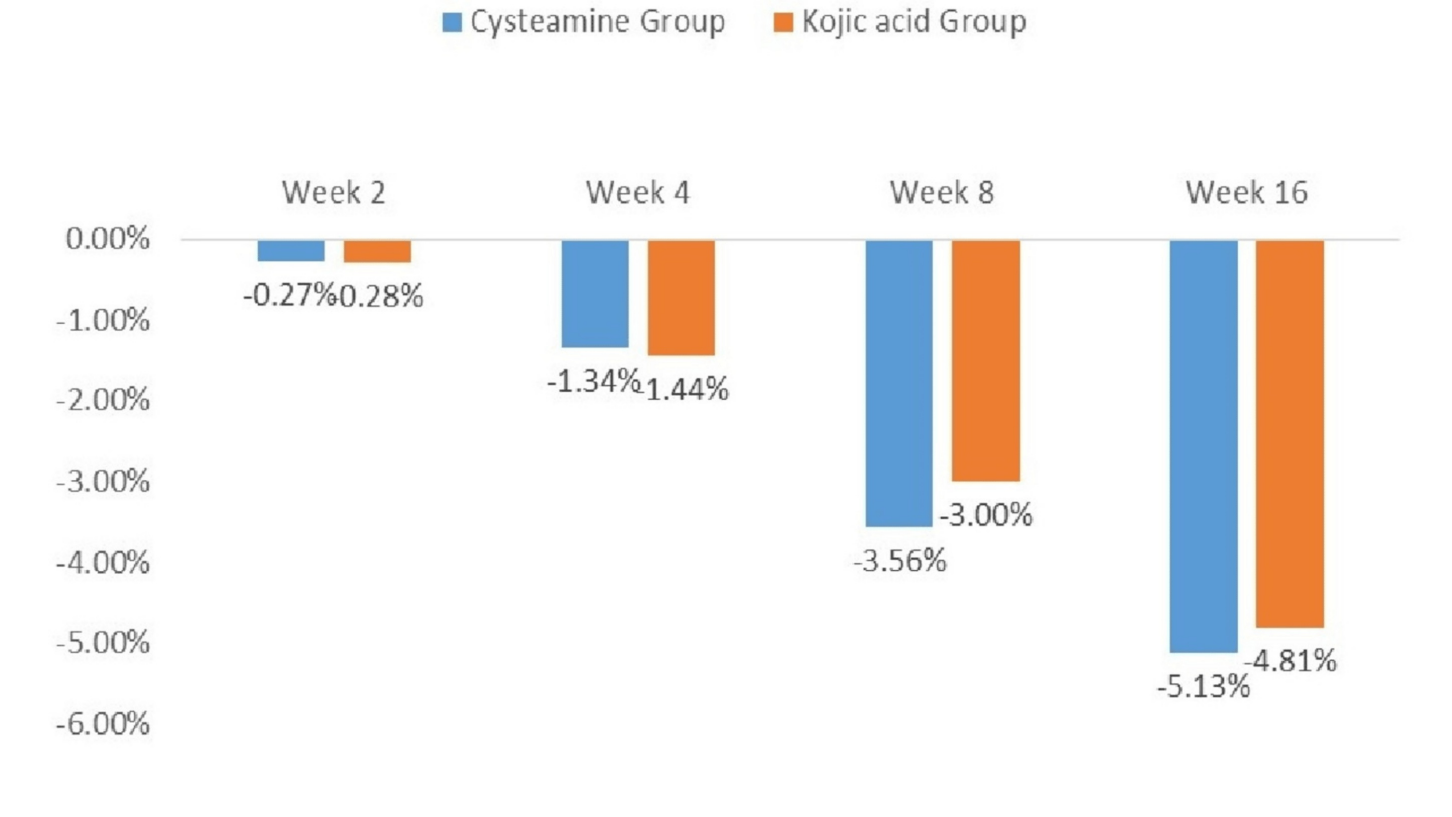
Percentage change in mean melanin content compared to baseline.

A statistically significant difference in mean melanin content between the lesions and surrounding normal areas was observed in both groups at week 16 compared to baseline, as measured by the Mexameter. Changes in mean Mexameter scores at each follow-up visit for both groups are shown in Table 3 and Figure 3.

**Table 3:** Changes in Mexameter score in both groups during the study period. ^+^Wilcoxon signed-rank test, statistically significant at p < 0.05. ^++^Wilcoxon signed-rank test, highly significant at p < 0.001. AU: arbitrary units.

Visit	Cysteamine group (n = 36)	Kojic acid group (n = 34)
Baseline (AU, mean ± SD)	468.55 ± 100.71	468.00 ± 84.96
Week 2 (AU, mean ± SD)	467.30 ± 100.81^++^	466.68 ± 84.89^++^
Week 4 (AU, mean ± SD)	462.27 ± 101.58^+ +^	461.27 ± 84.74^++^
Week 8 (AU, mean ± SD)	453.90 ± 105.50^++^	453.94 ± 86.25^++^
Week 16 (AU, mean ± SD)	446.56 ± 104.59^++^	445.51 ± 86.55^++^

**Figure 4:**
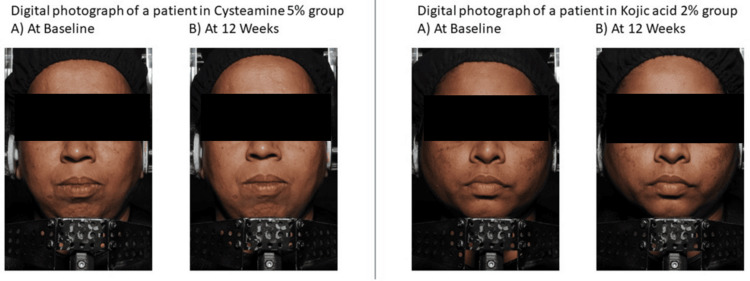
Digital photographs of patients from both groups at baseline and 12 weeks.

In the cysteamine group, the mean melanin content in lesions was reduced by 24.02 AU (5.13%) at week 16 compared to baseline (446.56 AU ± 104.59 AU vs. 468.55 AU ± 100.71 AU; p < 0.001). In the kojic acid group, the mean melanin content in lesions was reduced by 22.49 AU (4.81%) at week 16 compared to baseline (445.51 AU ± 86.55 AU vs. 468.00 AU ± 84.96 AU; p < 0.001), as shown in Figure 3. There was no statistically significant difference in melanin content reduction in lesions between the groups at any time point. Figure 4 shows digital photographs of patients from both groups at baseline and week 12.

Safety evaluation

There were no occurrences of clinical signs (i.e., erythema, edema, dryness, peeling, and scaling) reported by the investigator or functional signs (i.e., itching and tingling) reported by the patients during the study period.

Subject’s self-evaluation of effectiveness and product acceptability

Both drugs were well-received by all patients for their appealing color, non-sticky consistency, quick skin absorption, and spreadability. None of the patients reported itching, burning, or irritation with either product. There were no complaints about the smell, and product acceptability was high for both treatments. Based on self-evaluation, all patients in both groups reported improvements in their melasma during the study period.

## Discussion

Since its introduction by Kligman and Willis in 1975, the "triple" combination therapy (TC), which includes 5% hydroquinone + 0.1% tretinoin + 0.1% dexamethasone, along with various modifications, has remained a popular topical treatment for melasma, a common hyperpigmentation condition [17]. However, the undesirable side effects of these chemicals have sparked interest in alternative depigmenting agents such as cysteamine and kojic acid [17].

Kojic acid is a well-known depigmenting agent widely used in melasma treatments, with numerous clinical studies supporting its efficacy and safety [15,16]. On the other hand, cysteamine has only recently been introduced in India. This study aimed to compare the effectiveness and safety of 5% cysteamine and a combination of 2% kojic acid 2% and vitamin C in melasma patients.

Cysteamine, the simplest aminothiol produced in mammalian cells, is widely recognized for its antioxidant properties. It has also demonstrated anti-carcinogenic and anti-mutagenic effects [1,18,19]. Although the exact mechanism of cysteamine in inhibiting melanogenesis is not fully understood, it is believed to work through several pathways, including inhibiting tyrosinase and peroxidase, scavenging dopaquinone, chelating iron and copper ions, and increasing intracellular levels of glutathione, which regulates melanin production [18,19]. Kojic acid, a fungal product that is hydrophilic in nature, is useful in hyperpigmentation disorders like melasma, due to its inhibitory action on tyrosinase production and also serves as a potent antioxidant [20,21]. Despite similar targets, differences in formulation, penetration, and melanogenesis modulation may contribute to the variation in observed outcomes [19]. While both are commonly prescribed for the medical management of melasma, a recent review suggested reducing the use of kojic acid-containing topicals due to concerns over its safety profile, keeping currently available evidence in mind [22].

In our study, both treatment groups showed a statistically significant improvement in mMASI scores from baseline to the end of the therapy. However, the differences between the groups were not statistically significant, indicating that both drugs were equally effective in treating melasma. The reduction in melanin content also showed similar results, though the cysteamine group demonstrated numerically better outcomes than the kojic acid group. Both treatments were well tolerated, with no adverse events reported. To the best of our knowledge, this is the first head-to-head comparison between 5% cysteamine cream and 2% kojic acid cream in the medical management of melasma in Indian patients.

Two randomized controlled trials (RCTs) evaluated kojic acid in melasma treatment. In one 80-patient single-blind RCT, four different formulations of 1% kojic acid, either alone or combined with 2% hydroquinone and/or 0.1% betamethasone, were tested. All four groups significantly reduced mMASI scores after 12 weeks of daily treatment, with the best results seen in the 1% kojic acid and 2% hydroquinone groups. However, 1% kojic acid with 0.1% betamethasone was the least effective, and the combination of 1% kojic acid, 2% hydroquinone, and 0.1% betamethasone was associated with acneiform eruptions [16].

In contrast, six well-designed double-blind RCTs demonstrated that cysteamine significantly reduced mMASI scores compared to placebo and standard therapy [23-28]. The severity of melasma before and after treatment was assessed using the mMASI score across all studies. Some studies also employed colorimetric tools like the Mexameter® and Dermacatch® (Colorix, Neuchatel, Switzerland) to quantitatively analyze skin color reduction in treated areas. These reflectance-based tools are sensitive enough to distinguish skin pigmentation changes from side effects such as erythema [29]. While these methods are more precise, the mMASI score remains relevant as it visually assesses hyperpigmentation, a critical factor in aesthetic care. Across all studies, continuous and progressive depigmentation was observed over the four-month evaluation period. Cysteamine was well tolerated, with minimal adverse events reported. Additionally, two review papers supported cysteamine's effectiveness, noting a low likelihood of side effects [1,18]. Moreover, Austin et al. have documented a weak recommendation for kojic acid when combined with other agents and a strong recommendation for cysteamine as a stand-alone agent, as it had a beneficial efficacy and safety profile [19]. A 5% cysteamine cream may be preferred in patients seeking a non-HQ alternative with a potential for long-term use [18,19,23]. Additionally, its favorable safety profile, as highlighted in recent expert reviews, supports its role as a desirable topical therapy for melasma [19].

There were several limitations to the study. Firstly, the open-label study design may introduce bias in subjective measures like mMASI scores and patient-reported evaluations. To mitigate this, the same dermatologist performed the assessments to ensure consistency. Secondly, the sample size was relatively small and not powered to detect small but clinically meaningful differences between treatment groups. Thirdly, the follow-up period was limited to 16 weeks in the present study. Fourthly, there were differences in the application protocols: the short contact nature of the 5% cysteamine cream versus the overnight application of the 2% kojic acid cream may have influenced the treatment outcomes independently of the active ingredients. Future studies should aim to overcome the aforementioned limitations by incorporating a larger sample size, a blinded study design, with independent outcome assessors, and long-term follow-up to compare the long-term effectiveness, safety, and recurrence rates. Additionally, stratification of participants based on the type of melasma (epidermal, dermal, mixed), Fitzpatrick skin type, baseline severity, and prior treatment history will help better understand the differing treatment responses. Finally, standardizing application protocols or using matched contact times could help isolate the effect of the active ingredient.

## Conclusions

Both groups showed good efficacy, with statistically significant reductions in mean mMASI scores and melanin content in lesions, as measured by the Mexameter, compared to baseline. At week 16, 5% cysteamine cream demonstrated a clinically significant greater mean reduction in mMASI score (0.87, 12.25%) and melanin content (24.02 AU, 5.13%), compared to the kojic acid group (0.78, 10%; 22.49 AU, 4.81%), although no statistically significant difference was observed between the groups. Both treatments were well tolerated, with no adverse events reported during the study. 

Five percent cysteamine cream is a well-tolerated and effective alternative for melasma management, demonstrating comparable efficacy to 2% kojic acid cream over 16 weeks. Therefore, 5% cysteamine cream is a valuable addition to the medical management of melasma.

## Appendices

Appendix A

**Table 4:** Subject's self-evaluation questionnaire

Domain	Questionnaire items	1 = completely agree	2 = somewhat agree	3 = somewhat disagree	4 = completely disagree
Product effectiveness	The test product helps reduce melasma				
Physical characteristics	The color of the product is appealing				
	The test product is quickly absorbed into the skin				
	The test product does not leave the skin sticky				
	The test product spreads properly on the face				
Product acceptability	The test product does not cause itching				
	The test product does not cause irritation				
	The test product does not give a burning sensation				
